# Patterns, management options and outcome of blunt thoracic aortic injuries: a 20-year experience from a Tertiary Care Hospital

**DOI:** 10.1007/s00068-022-01930-1

**Published:** 2022-03-14

**Authors:** Hassan Al-Thani, Suhail Hakim, Mohammad Asim, Kaleem Basharat, Ayman El-Menyar

**Affiliations:** 1 Trauma and Vascular Surgery Section, Hamad Medical Corporation, PO Box 3050, Doha, Qatar; 2 Trauma Surgery Section, Hamad General Hospital (HGH), Doha, Qatar; 3 Clinical Research, Trauma and Vascular Surgery Section, HGH, Doha, Qatar; 4 Emergency Department, HGH, Doha, Qatar; 5 Clinical Medicine, Weill Cornell Medical College, Doha, Qatar

**Keywords:** Thoracic aortic injury, Blunt trauma, Thoracic endovascular aortic repair, Open surgery, Outcome

## Abstract

**Background:**

Blunt Thoracic aortic injury (BTAI) is the second leading cause of mortality after head injuries in blunt trauma patients. There is a paucity of information on the presentation, management and outcome of BTAIs from the Middle Eastern region. We explored the patterns, management options and outcomes of BTAIs in a level I trauma center.

**Methods:**

We conducted a retrospective observational study on all adult patients who were admitted with BTAIs between 2000 and 2020. Patients were compared for the management option (conservative *vs* endovascular aortic repair (TEVAR) *vs* open surgery) and outcomes. Comparison between the respective groups was performed using one-way analysis of variance for continuous variables, and Pearson chi-square test for categorical variables. Kaplan–Meier curve and Cox regression analysis were performed for the outcome.

**Results:**

Eighty-seven patients had BTAI (82% male) with mean age 37.3 ± 14.5 years. The mean injury severity score was 30 ± 10 and the aortic injury grade was III (I–IV). Grade III (41.4%) and Grade IV (33.3%) injuries were more common followed by Grade II (13.8%) and Grade I (11.5%). Forty percent of cases were treated conservatively whereas aortic interventions were performed in 60% of cases (*n* = 52). The TEVAR was performed in 33 patients (63.5%), and 19 (36.5%) were treated with open surgery (14 with graft interposition and 5 with clamp and direct repair). The aortic injury grade was significantly higher in the intervention groups as compared to the conservative group (*p* = 0.001). Patients with Grade IV injuries were more likely to be treated by open repair whereas a higher frequency of patients with grade III was managed by TEVAR (*p* = 0.001). All the patients with Grade I–II were treated conservatively. The overall in-hospital mortality rate was 25.3% and it was significantly higher in the conservative group (40.0%) in comparison to the open repair (31.6%) and TEVAR (6.1%) group (*P* = 0.004). More of the non-survivors sustained head injuries (*P* = 0.004), had higher ISS (*P* = 0.001) and greater aortic injury grades (*P* = 0.002), and were treated non-operatively (*P* = 0.001).

**Conclusions:**

BTAI seems not common in trauma, however, one quarter of cases died in a level 1 trauma center, prehospital deaths were not analyzed, and postmortem examination was lacking. The associated head injury and aortic injury grade have an impact on the management option and hospital outcome. The conservative and TEVAR options were performed almost equally in 78% of cases. TEVAR and open surgery were performed only for aortic injury grade III or IV whereas the conservative treatment was offered for selected cases among the 4 injury grades. However, the mortality was higher in the conservative followed by the open surgery group and mostly due to the associated severe head injury. TEVAR should be considered for patients requiring intervention unless contraindicated due to technical difficulties. Appropriately selected patients with low-grade injuries may be managed conservatively. Long-term follow-up is needed in young adults for concerns of aortic remodeling and complications.

## Introduction

Blunt Thoracic aortic injury (BTAI) is the second leading cause of mortality in patients who sustained blunt traumatic injuries, after severe head trauma involving intracranial hemorrhage [1]. However, aortic injuries are infrequent, only accounting for 1.5% to 2% of thoracic trauma but are potentially fatal [2–4]. The common injury mechanisms associated with BTAI include road traffic accidents, pedestrian injuries, fall from height, and crush injuries [5, 6]. Early diagnosis of BTAI is challenging due to the presence of concomitant life-threatening injuries in patients with severe thoracic trauma [7]. Clinical suspicion, hemodynamic stability, availability, and speed of access to imaging modalities are all part of the diagnostic procedure for BTAI [8]. Notably, clinical symptoms such as systemic hypotension, upper limb hypertension, asymmetry of limb pulses, and flow murmurs are not diagnostically trustworthy [9]. Therefore, computed tomographic angiography (CTA) is the diagnostic modality of choice for hemodynamically stable BTAI [1]. Transesophageal echocardiography (TEE) is frequently used to guide surgical decisions in unstable polytrauma patients because it may be conducted at the bedside or intraoperatively [10].

A usually higher proportion of BTAI involves the proximal descending aorta (54–65%) followed by the ascending aorta or arch (10–14%), mid to distal descending aorta (12%) and multiple sites (13–18%) [11]. Traumatic aortic injuries are broadly classified into four grades depending on injury severity, such as Grade I: intimal tear, Grade II: intramural hematoma, Grade III: pseudoaneurysm, and Grade IV: free rupture [12]. The Grade II-IV injuries are often assessed clinically to look for the need of surgical repair. Nonoperative (conservative) management is well established for grade I injuries, including aggressive anti-impulse therapy (controlling the heart rate and blood pressure medically to minimize the wall shear stress and propagation) with inpatient monitoring and surveillance imaging [13]. Currently, the therapeutic paradigm for BTAI has been shifted toward endovascular repair. Thoracic endovascular aortic repair (TEVAR) has become the treatment of choice due to decreased mortality and less procedural complications than open aortic repair [1, 14, 15]. On the other hand, open repair with anticoagulation is associated with a significant mortality risk ranging from 24 to 42%. Earlier studies have reported the incidence, mechanism of injury, and management of BTAIs from different parts of the world. [2, 7, 9, 16]. However, there is a paucity of information on the BTAIs in the Middle East. This study aimed to analyze the patterns of aortic injury, and management options and outcomes of BTAI in the level I trauma patients in Qatar over 20 years.

## Methods

A retrospective observational study was performed to review data for all consecutive patients with BTAI admitted and treated at the only level I Trauma Center in Qatar between 2000 and 2020. Data were retrieved from the National Trauma Registry database and electronic medical records (CERNER). All adult BTAI patients admitted to the vascular surgery department at HGH were included in the study. Patients aged < 18 years, who sustained penetrating injuries, prehospital or on-arrival deaths were excluded from the study.

Data included patient demographics (age, gender, nationality), mechanism of injury, initial vital signs at scene and emergency department, associated injuries, Injury severity score (ISS), abbreviated injury scores (AIS), Aortic injury score, thoracotomy, exploratory laparotomy, type of aortic operation (clamp and direct repair, graft interposition and endovascular aortic stent), adjuncts used, complications (paraplegia due to trauma, paraplegia due to ischemia, deep vein thrombosis (DVT) and pulmonary embolism(PE)), ventilatory days, ICU length of stay (LOS), hospital LOS, in-hospital mortality, cause of death, follow-up imaging and duration of clinical follow-up.

**Fig. 1:**
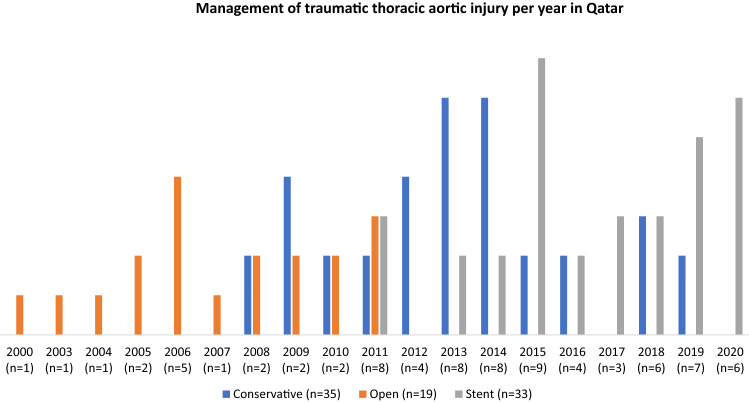
The 3 management options of BTAI per year

The diagnosis of BTAI was confirmed by radiography (Contrast-enhanced spiral computed tomography or aortography) or during operative exploration. The BTAI were classified into four categories (Grade I to IV) based on the severity of aortic injury and location as described by Azizzadeh et al. [12]. The BTAI patients were evaluated by a multidisciplinary team comprised of trauma surgeons, radiologists, vascular surgeons and, if needed, cardiothoracic surgeons. The clinical decision making to choose either non-operative management or surgical approach was based on the patient’s clinical condition, grade, aortic size and location of the aortic injury. All patients who received ATLS management protocol on arrival and including those treated non-operatively, were admitted to the Trauma ICU for close monitoring for hemodynamic stability and complications such as delayed rupture and bleeding, definitive treatment for the aorta, and management of concomitant injuries. The operative management mainly constituted open repair or Endovascular repair (TEVAR). During the last 20 years of the study, there was a significant transformation of trauma care. Before 2007 trauma care was under general surgery and admission management wise by the emergency physician. Emergency registry was not well established with minimum data set that include only the emergency department encounter and no intra-hospital or prehospital data. Following the establishment of the trauma center in 2007, there was an organized management of the trauma patient with protocols and guidelines. The trauma registry was established with a detailed data from prehospital all the way to rehabilitation and was linked to the national trauma database bank (NTDB) in the USA in 2012. The management of a traumatic aortic injury went into phases (Fig. 1): open repair depending only on the operative findings between 2000 and 2007, Open repair until 2011 was under trauma service. Endovascular repair protocol and guidelines were started at the end of 2011 for all cases that need intervention; but only one patient underwent open repair (this patient was hemodynamically unstable and endovascular stent size was not available). Expansion of the operating theater (OR) with the establishment of a hybrid operating room was in 2016. The Hybrid OR offers all interventions required for unstable patients in the same setting (including abdominal bleeding and neurosurgery assessment) without the need to transfer unstable patients between the OR and the angiography suite that resulted in a reduction of time to intervene.

### Open surgical repair

Open surgery was performed through a left postero-lateral thoracotomy approach using single lung ventilation with or without surgical adjuncts which comprised of distal perfusion with or without CSF drainage.

### Endovascular repair (Stent / TEVAR group)

All BTAI patients underwent preoperative imaging by a contrast CT angiography of the torso to evaluate the anatomy, morphology, grade of injury of the aorta and suitability of TEVAR. The procedures were performed in the hybrid operating room with a backup plan for open repair. Open approach was used to access the common femoral artery in all the cases with the exception of one female patient who required access through the right iliac artery due to a small femoral artery to introduce the sheath or endograft to minimize complications. We obtained second vascular access through the left brachial artery using percutaneous ultrasound guidance. A 5-French introducer sheath and a pigtail catheter passed over 0.035-inch guide wire into the ascending thoracic aorta. The Automated contrast injection system is attached to the pigtail catheter to perform the arch arteriogram. The pigtail catheter is also used as a marker for the left subclavian artery during the deployment of the endograft. We used the Medtronic Valiant Captivia thoracic stent graft system in all the cases. Notably, the time to aortic intervention primarily depends on the patient general condition, the severity of BTAI, risk of bleeding and associated traumatic brain injury (TBI). The follow-up contrast-enhanced spiral CT scan was performed at 6, 12 months and later annually.

### Statistical analysis

Data were reported as proportion, mean (± standard deviation), median, and range, when applicable. Patients were classified based on the type of management (“Conservative group”, “Open surgery group”, and “Stent group (TEVAR)”) and outcome (survived *vs* deceased). Comparison between the respective groups was performed using one-way analysis of variance (ANOVA) for continuous variables, and Pearson’s chi-square test was used to compare proportions between the groups for categorical variables. A significant difference was considered when the 2-tailed *p* value was less than 0.05. The Kaplan–Meier curve was constructed to display the survival analysis based on the thoracic aortic injury management option. Cox regression analysis was performed to assess the hazard ratio (HR) and 95% CI for the risk of mortality during the follow-up based on the type of aortic management after adjusting for sex, age, head injury, ISS, GCS at ED, and aortic injury score. Patients were censored at the time of death or lost to follow-up. Data analysis was carried out using the Statistical Package for Social Sciences version 21 (SPSS Inc, Chicago, Illinois).

## Results

During the 20-year study period, 87 trauma patients were diagnosed with BTAI, of which 3 (3.4%) had concomitant abdominal aortic injuries, which were small intimal flap treated conservatively. Figure 1 shows the 3 management options of BTAI per year. After 2011, BTAI was treated with the conservative or TEVAR option only. Grade IV aortic injury was found in 50% (16/32) of cases treated between 2000 and 2011 and 24% (13/55) of cases between 2012 and 2020 there was no open surgery done after 2011.

Table 1 summarizes the overall patients’ demographics, clinical characteristics, management, and outcome. The mean age was 37.3 ± 14.5 years, and 82% were males (Road traffic accidents (64.4%) was the most frequent mechanism of injury followed by auto–pedestrian accident (14.9%) and fall from height (12.6%). The frequent mode of transportation of injured patients from the scene to the hospital was ground ambulance (83%), whereas 13% were transferred by the EMS Helicopter for distant locations. The mean vital signs at the scene of injury and in the ED were within normal limits. The mean injury severity score (ISS) was 30.3 ± 10.2, chest AIS was 4.1 ± 0.4 and head AIS was 3.9 ± 0.9. The median aortic injury grade was III (I–IV), of which Grade III (41.4%) and Grade IV (33.3%) injuries were more common followed by Grade II (13.8%) and Grade I (11.5%).

**Table 1:** Demographics, clinical characteristics, management, and outcome of patients with blunt thoracic aortic injuries (*n* = 87)

Variable	Value	Variable	Value
Age (mean ± SD) years	37.3 ± 14.5	Mean Abdomen AIS	2.6 ± 0.8
Gender		Mean Spine AIS	2.2 ± 0.6
Female	16 (18.4%)	Mean Upper extremity AIS	2.0 ± 0.3
Male	71 (81.6%)	Mean Pelvis AIS	2.4 ± 0.6
Mechanism of Injury		Mean Lower extremity AIS	2.6 ± 0.5
Road traffic accidents	56 (64.4%)	Aortic injury score (median range)	3 (1–4)
Pedestrian	13 (14.9%)	Grade I	10 (11.5%)
Fall from height	11 (12.6%)	Grade II	12 (13.8%)
Fall of heavy object	6 (6.9%)	Grade III	36 (41.4%)
Self-inflected	1 (1.1%)	Grade IV	29 (33.3%)
Mode of Transportation		Thoracotomy	20 (23.0%)
Ground EMS	72 (82.7%)	Exploratory laparotomy (*n* = 65)	13 (20%)
Helicopter EMS	11 (12.8%)	Aortic interventions	52 (59.8%)
Private	4 (4.7%)	Clamp and direct repair	5 (9.6%)
Scene SBP	121.2 ± 32.7	Graft interposition	14 (26.9%)
Scene DBP	77.5 ± 23.9	Endovascular aortic stent	33 (63.5%)
Scene Pulse	96.9 ± 26.6	Adjuncts	12 (13.8%)
Scene respiratory rate	20.1 ± 7.6	Passive bypass	1 (2.0%)
Scene oxygen saturation	95.5 ± 5.5	Atrio-femoral bypass*	8 (15.7%)
Scene GCS	15 (3–15)	Traditional cardiopulmonary bypass	3 (5.9%)
SBP at ED	119.2 ± 23.0	Paraplegia due to trauma	3 (3.4%)
DBP at ED	73.9 ± 17.7	Paraplegia due to ischemia	1 (1.1%)
Pulse rate at ED	101.1 ± 19.1	Deep vein thrombosis	1 (1.1%)
Respiratory rate at ED	20.9 ± 4.6	Pulmonary embolism	2 (2.3%)
Oxygen saturation at ED	97.9 ± 3.1	Ventilatory days	4 (1–23)
GCS at ED	15 (3–15)	ICU length of stay (days)	6 (1–58)
Abdominal Aorta injury	3 (3.4%)	Hospital length of stay (days)	11 (1–146)
Head injury (*n* = 76)	22 (28.9%)	In-hospital mortality	22 (25.3%)
Chest injury (*n* = 76)	76 (100%)	Cause of death	
Abdominal injury (*n* = 76)	40 (52.6%)	Bleeding	5 (22.7%)
Sternal fracture	15 (19.7%)	Traumatic brain injury (TBI)	11 (50.0%)
Diaphragm injury (*n* = 76)	2 (2.6%)	Bleeding and TBI	6 (27.3%)
Solid organ injury (*n* = 76)	31 (40.8%)	Follow-up imaging	51 (58.6%)
Mesenteric injury (*n* = 76)	7 (9.2%)	Duration of follow-up (days)	244 (1–6328)
Mean Injury severity score	30.3 ± 10.2		
Mean Chest AIS	4.1 ± 0.4		
Mean Head AIS	3.9 ± 0.9		

*ED* emergency department; *using centrifugal pump (without heparinization); ** with total body heparinization, *AIS* abbreviated injury score; *GCS* Glasgow coma scale

**Fig. 2:**
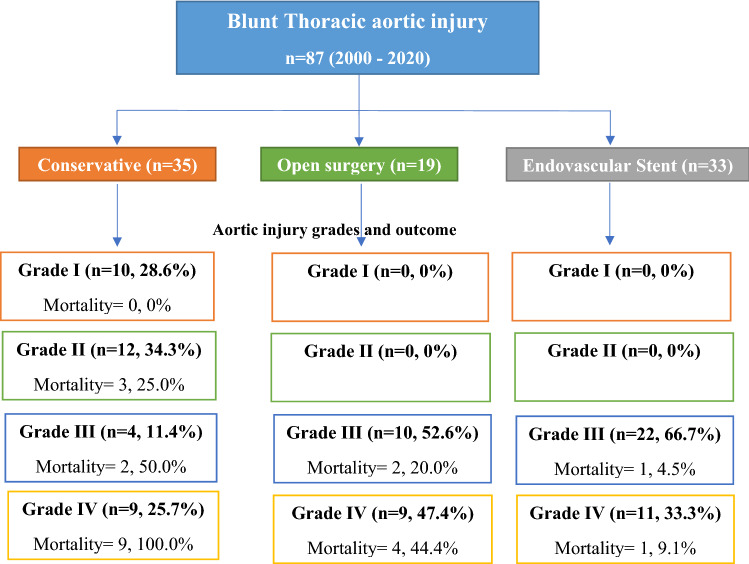
Study design and outcomes

Forty percent of cases (*n* = 35) were treated conservatively whereas aortic interventions were performed in 60% of cases (*n* = 52). The TEVAR was performed in 33 patients, and 19 were treated with open repair operations (14 with graft interposition and 5 with clamp and direct repair) (Fig. 2). Adjuncts were required in only 12 patients in the open repair group: three patients who underwent open repair with distal perfusion using traditional cardiopulmonary bypass had full heparinization, eight patients had atrio-femoral bypass using a centrifugal pump, and one had passive bypass shunts without heparinization.

Three patients had paraplegia due to trauma, one had paraplegia due to ischemia, two developed PE, and one had DVT. The patient with paraplegia secondary to spinal cord ischemia underwent an emergency open repair for grade 4 injury. The patient was unstable, and the procedure was done without adjunct (i.e. there was no distal perfusion and no CSF drainage); only clamp and repair using interposition graft. The aortic clamp time was 54 min. Postoperatively, CSF drainage and steroid intravenous were tried without any improvement. The overall in-hospital mortality rate was 25.3% (*n* = 22), and 11 patients died due to TBI, 6 had severe bleeding plus TBI and the remaining 5 deaths were attributed to bleeding. The follow-up imaging was done in 51(78.5%) patients and the median duration of clinical follow-up was 244 (1–6328) days.

Tables 2 and 3 demonstrate the patients’ characteristics, complications and outcomes based on management approach, i.e. non-operative treatment (40.2%), endovascular aortic repair (TEVAR; 37.9%) and open surgery (21.8%). The three groups were comparable for age, mechanism of injury, mode of transportation, initial vital signs at ED, the severity of the injury, and associated injuries except for gender. Males were more likely to be treated conservatively, whereas females had more open surgeries (*P* = 0.001). The median aortic injury grade was significantly higher in the open repair and TEVAR group as compared to the conservative group (*p* = 0.001). Patients with Grade IV injuries were more likely to be treated by open repair whereas a higher frequency of patients with grade III was managed by stenting (*p* = 0.001). All the patients with Grade I and II were treated conservatively. Notably, 4 patients in grade III and nine with grade IV were managed conservatively mainly because of associated severe head injury.

**Table 2:** Comparison of demographics and clinical characteristics by management approach

	Conservative group (*n* = 35; 40%)	Open group (*n* = 19; 22%)	Stent group (*n* = 33; 38%)	*P* value
Age (mean ± SD) years	40.6 ± 16.4	37.1 ± 10.9	34.1 ± 13.8	0.19
Male	34 (97.1%)	9 (47.4%)	28 (84.8%)	0.001
Females	1 (2.9%)	10 (52.6%)	5 (15.2%)	
Mechanism of injury				
Traffic-related	21 (60.0%)	17 (89.5%)	18 (54.5%)	0.11 for all
Pedestrian	7 (20.0%)	2 (10.5%)	4 (12.1%)	
Fall from height	3 (8.6%)	0 (0.0%)	8 (24.2%)	
Fall of heavy object	3 (8.6%)	0 (0.0%)	3 (9.1%)	
Self-inflected	1 (2.9%)	0 (0.0%)	0 (0.0%)	
Mode of transportation				
Ground EMS	31 (88.2%)	16 (84.2%)	25 (75.8%)	0.47 for all
Helicopter EMS	3 (8.8%)	3 (15.8%)	5 (15.2%)	
Private	1 (2.9%)	0 (0.0%)	3 (9.1%)	
SBP at ED	122.5 ± 26.9	112.0 ± 18.8	117.3 ± 19.4	0.47
DBP at ED	75.0 ± 19.4	69.1 ± 10.7	74.0 ± 17.5	0.73
Pulse rate at ED	98.7 ± 18.6	111.6 ± 29.6	101.3 ± 16.8	0.27
Respiratory rate at ED	19.9 ± 3.7	21.4 ± 3.5	21.7 ± 5.4	0.31
Oxygen saturation at ED	97.3 ± 3.7	99.0 ± 1.5	98.2 ± 2.7	0.35
GCS ED; median (range)	15 (3–15)	15 (8–15)	15 (3–15)	0.13
GCS ED; mean (95% CI)	10.6 (8.7–12.5)	13.9 (11.8–15.9)	13.1 (11.6–14.5)	0.13
Head (*n* = 76)	13 (37.1%)	1 (12.5%)	8 (24.2%)	0.27
Abdomen	18 (51.4%)	3 (37.5%)	19 (57.6%)	0.58
Injury severity score	31.4 ± 11.6	25.5 ± 5.3	30.2 ± 9.4	0.34
Head AIS	4.2 ± 0.8	3.0 ± 0.0	3.4 ± 0.8	0.08
Chest AIS	4.0 ± 0.4	4.3 ± 0.5	4.1 ± 0.4	0.27
Abdomen AIS	2.6 ± 0.9	2.3 ± 0.6	2.5 ± 0.8	0.86
Aortic injury grade (median range)	2 (1–4)	3 (3–4)	3 (3–4)	0.001
I	10 (28.6%)	0 (0.0%)	0 (0.0%)	0.001 for all
II	12 (34.3%)	0 (0.0%)	0 (0.0%)	
III	4 (11.4%)	10 (52.6%)	22 (66.7%)	
IV	9 (25.7%)	9 (47.4%)	11 (33.3%)	

ED: Emergency department; AIS: abbreviated injury score, CI: confidence interval

**Table 3:** Complications and outcomes by management approach (*n* = 87)

	Conservative group (*n* = 35)	Open group (*n* = 19)	Stent group (*n* = 33)	*p* value
Thoracotomy	1 (2.9%)	19 (100%)	0 (0.0%)	0.001
Exploratory laparotomy (*n* = 65)	7 (20.0%)	1 (5.3%)	5 (15.2%)	0.34
Adjuncts	–	12 (63.2%)	0 (0.0%)	0.001
Passive bypass	–	1 (8.3%)	0 (0.0%)	0.001 for all
Atrio-femoral bypass using centrifugal pump (without heparinization)	–	8 (66.7%)	0 (0.0%)	
Traditional cardiopulmonary bypass (with total body heparinization)	–	3 (25.0%)	0 (0.0%)	
Paraplegia due to trauma	1 (2.9%)	1 (5.3%)	1 (3.0%)	0.88
Paraplegia due to ischemia	0 (0.0%)	1 (5.3%)	0 (0.0%)	0.16
Deep vein thrombosis	0 (0.0%)	1 (5.3%)	0 (0.0%)	0.16
Pulmonary embolism	1 (2.9%)	0 (0.0%)	1 (3.0%)	0.75
Ventilatory days	4.5 (1–20)	2 (1–16)	4.5 (1–23)	0.64
ICU length of stay (days)	5 (1–58)	15 (2–38)	8.5 (3–39)	0.02
Hospital length of stay (days)	6 (1–91)	16 (1–78)	27 (3–146)	0.001
Mortality (*n* = 22)	14 (40.0%)	6 (31.6%)	2 (6.1%)	0.004
Cause of death				
Bleeding	2 (14.3%)	3 (50.0%)	0 (0.0%)	0.41 for all
Traumatic brain injury (TBI)	7 (50.0%)	2 (33.3%)	2 (100%)	
Bleeding and TBI	5 (35.7%)	0 (0.0%)	0 (0.0%)	
Follow-up imaging	17 (48.6%)	8 (42.1%)	26 (78.8%)	0.01
Duration of follow-up (days)	83 (1–4255)	186 (1–6328)	345 (2–2228)	0.08
Mortality*(*n* = 5)	2 (5.7%)	3 (15.8%)	0 (0.0%)	0.06

After excluding head injuries-related deaths using Yates corrected chi square for comparison between conservative and open surgery (*p* = 0.44)

Overall, thoracotomy was performed in 20 (23.0%) patients; 19 for the open surgery repair of the aortic injury and one for lung injury. Paraplegia due to ischemia (*n* = 1), DVT (*n* = 1) was observed in the open repair group, whereas PE occurred in two patients: one in the conservative and one in the Stent group (TEVAR).

Possibly due to careful selection of patients, there were no stent-related complications such as endoleak, migration, kinking, rupture or stroke. Intentional partial left subclavian artery coverage was done in 3 patients in the stent group, which was asymptomatic without revascularization during follow-up. No other stent-related complications were reported during the follow-up. No case was converted to an open repair and there were no reports of device-related aortic injury, including perforation or dissection.

In the endovascular group, full heparinization was used in selective patients, but the majorities were without heparin due to TBI or a high risk of bleeding. Patients in the open group had significantly prolonged ICU length of stay as compared to other groups (*P* = 0.02). Median hospital LOS for TEVAR was 27 days (range, 3–146 days) compared with open repair (16 days) and conservative group (6 days) (*P* = 0.001). The in-hospital mortality rate was significantly higher in the conservative group (40.0%) in comparison to the open repair (31.6%) and TEVAR (6.1%) group (*P* = 0.004). Patients in the TEVAR group were more likely to have follow-up imaging than others (*P* = 0.01).

Table 4 compares the characteristics of survivors and non-survivors. There were no differences in age, gender, initial vital signs, and in-hospital complications. More of the non-survivors sustained head injuries (*P* = 0.004), had higher ISS (*P* = 0.001) and aortic injury grades (*P* = 0.002), were treated conservatively (*P* = 0.001) and had significantly shorter hospital course (*P* = 0.001).

**Table 4:** Comparison of clinical characteristics, complications and management between the survivors and non-survivors

	Survived (*n* = 65)	Deceased (*n* = 22)	*P* value
Age (mean ± SD) years	37.1 ± 14.6	38.1 ± 14.7	0.80
Male	54 (83.1%)	17 (77.3%)	0.54
SBP at ED	120.4 ± 21.5	114.0 ± 29.1	0.37
Pulse rate at ED	99.9 ± 18.2	105.3 ± 22.3	0.32
GCS at ED	15 (3–15)	3 (3–15)	0.001
Head (*n* = 76)	12 (20.7%)	10 (55.6%)	0.004
Abdomen	31 (53.4%)	9 (50.0%)	0.79
Injury severity score	27.6 ± 8.1	38.9 ± 11.6	0.001
Head AIS	3.4 ± 0.8	4.6 ± 0.5	0.001
Chest AIS	4.1 ± 0.4	4.1 ± 0.3	0.60
Abdomen AIS	2.5 ± 0.7	2.9 ± 1.2	0.34
Aortic injury grades (median range)	3 (1–4)	4 (2–4)	0.002
I	10 (15.4%)	0 (0.0%)	0.003 for all
II	9 (13.8%)	3 (13.6%)	
III	31 (47.7%)	5 (22.7%)	
IV	15 (23.1%)	14 (63.6%)	
Thoracotomy	13 (20.0%)	7 (31.8%)	0.25
Exploratory laparotomy (*n* = 65)	7 (10.8%)	6 (27.3%)	0.06
Management			
Conservative	21 (32.3%)	14 (63.6%)	0.01 for all
Open surgery	13 (20.0%)	6 (27.3%)	
Endovascular aortic stent	31 (47.7%)	2 (9.1%)	
Adjuncts			
Passive bypass	0 (0.0%)	1 (12.5%)	0.77 for all
Atrio-femoral bypass using centrifugal pump (without heparinization)	5 (11.6%)	3 (37.5%)	
Traditional cardiopulmonary bypass (with heparinization)	3 (7.0%)	0 (0.0%)	
Paraplegia due to trauma	3 (4.6%)	0 (0.0%)	0.30
Paraplegia due to ischemia	1 (1.5%)	0 (0.0%)	0.55
Deep vein thrombosis	1 (1.5%)	0 (0.0%)	0.55
Pulmonary embolism	2 (3.1%)	0 (0.0%)	0.40
Ventilatory days	7 (1–23)	1.5 (1–8)	0.008
ICU length of stay (days)	7.5 (2–58)	3 (1–8)	0.002
Hospital length of stay (days)	20 (3–146)	3 (1–8)	0.001

Table 5 compares the aortic injury grade and outcome by type of management approach. Among the conservative group, all the patients with grade IV injuries had died whereas all grade I injury patients survived (*P* = 0.001). In the open surgery and TEVAR group, there was no significant difference in the outcome based on the aortic injury grades (Table 6).

**Table 5:** Comparison of aortic injury grade and outcome by type of management approach

Treatment group			*P* value
Conservative (*n* = 35)	Survived (*n* = 21)	Deceased (*n* = 14)	
Grade I	10 (47.6%)	0 (0.0%)	0.001 for all
Grade II	9 (42.9%)	3 (21.4%)	
Grade III	2 (9.5%)	2 (14.3%)	
Grade IV	0 (0.0%)	9 (64.3%)	

Treatment group			*P* value
Open surgery (*n* = 19)	Survivors (*n* = 13)	Non-Survivors (*n* = 6)	
Grade III	8 (61.5%)	2 (33.3%)	0.51 for all
Grade IV	5 (38.5%)	4 (66.7%)	

Treatment group			*P* value
Endovascular stent (*n* = 33)	Survivors (*n* = 31)	Non-Survivors (*n* = 2)	
Grade III	21 (67.7%)	1 (50.0%)	0.79 for all
Grade IV	10 (32.3%)	1 (50.0%)

**Table 6:** Cox proportional hazard analysis for predictors of BTAI mortality

Variables	Hazard ratio	95% CI		*P* value
		Lower	Upper	
Age	1.026	0.981	1.073	0.259
Sex	0.172	0.013	2.259	0.181
Head injury	0.312	0.031	3.105	0.320
Injury severity score	1.030	0.948	1.119	0.482
GCS at ED	0.805	0.683	0.949	0.010
Aortic injury score	3.675	1.482	9.111	0.005
Open surgery				0.026
Conservative treatment	2.805	0.211	37.344	0.435
TEVAR (stenting)	0.179	0.012	2.649	0.211

**Fig. 3:**
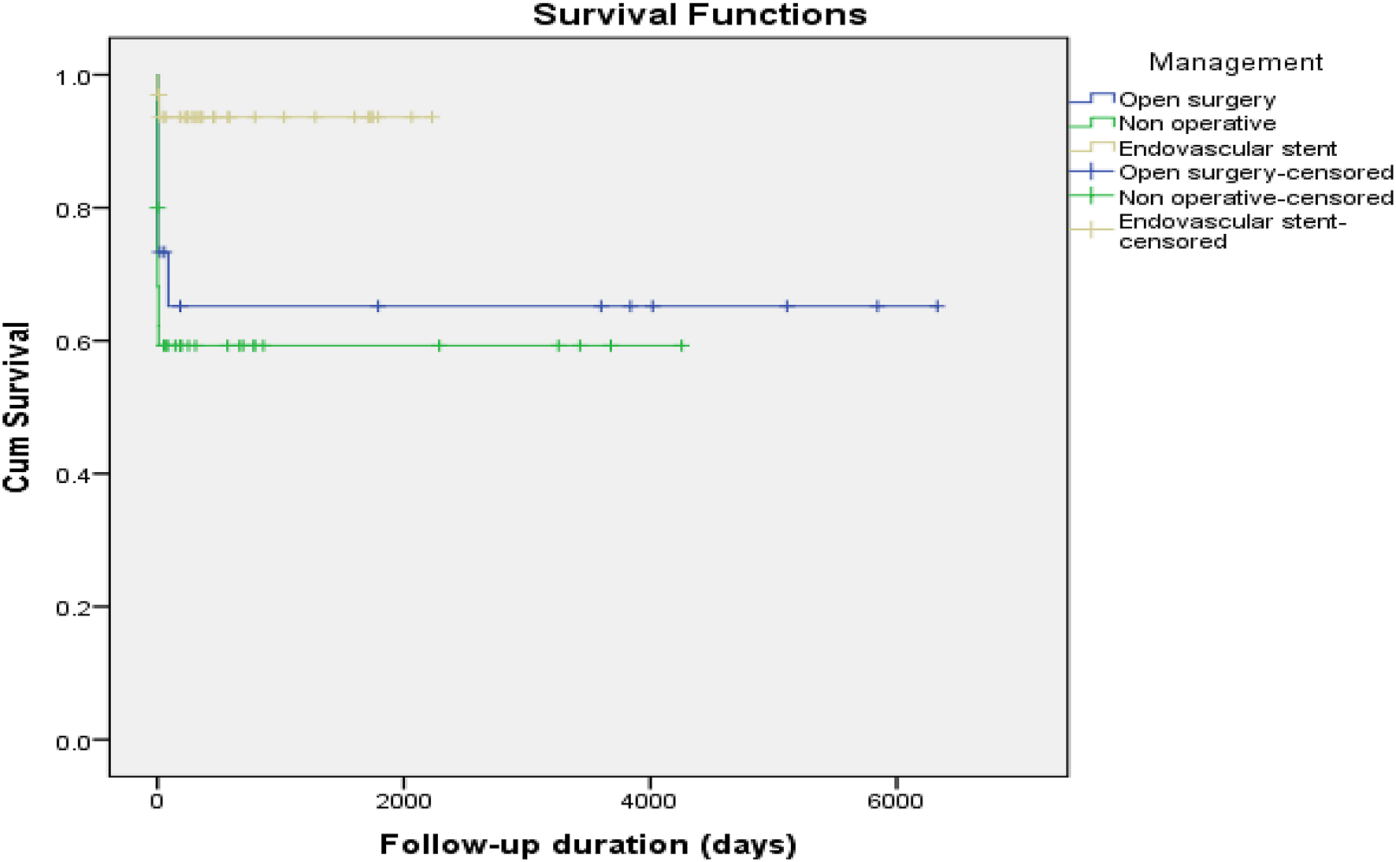
Kaplan–Meier survival analysis based on thoracic aortic injury management

**Fig. 4:**
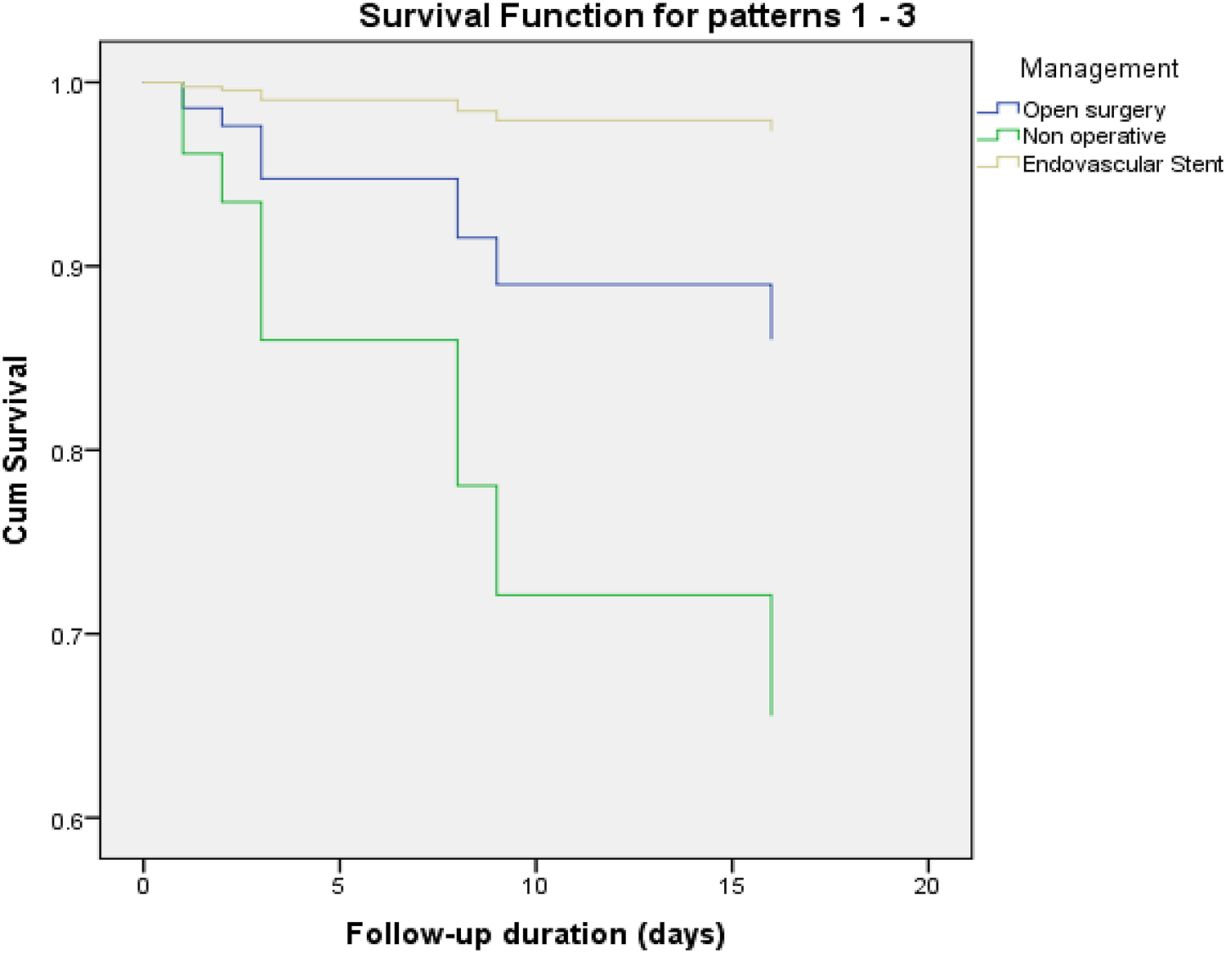
Cox proportional hazard model for potential risk factors affecting mortality

Based on thoracic aortic injury management, the duration of follow-up (days) for the open surgery group was significantly higher [4132 (95% CI 2559–5704)] as compared to the conservative [2415(95% CI 1687–3143)] and the TEVAR group 2081 (95% CI 1885–2277). Kaplan–Meier survival (log-rank test) showed significant difference with respect to mortality based on the management approaches i.e. non-operative (conservative), endovascular aortic repair (TEVAR) and open surgery (*P* = 0.003; Fig. 3). Moreover, after adjusting for age, sex, head injury, ISS, GCS at the ED, and aortic injury grade, the Cox regression model showed that GCS at the ED (HR: 0.805, 95% CI: 0.683–0.949, *P* = 0.010) and aortic injury grade (HR: 3.675, 95%CI: 1.482–9.111, *P* = 0.005) were independent predictors of mortality (Fig. 4).

## Discussion

This is a unique study from Qatar to analyze the management and outcome of BTAIs with long-term follow-up at the only Level I trauma center in the country over 20-year period. The study showed that 40% of BTAIs were managed conservatively while 60% required aortic interventions. The associated head injury and aortic injury grade influenced the management option and hospital outcome. The median aortic injury grade was significantly higher in the open repair and TEVAR groups as compared to the conservative group. Among the conservative group, patients with grade IV injuries died (with severe TBI), whereas grade I injury patients survived. In the open surgery and TEVAR group, there was no significant difference in the outcome based on the aortic injury grade. Non-operative management was the primary approach to the low-grade BTAI, yet the outcomes of this approach appear to be detrimental compared to any of the operative approaches. This could be due to more severe head injury in this group as 12 out of 14 patients died with severe head injuries. The mean GCS score and head AIS were worse in the conservative group but the difference was not statistically significant among the 3 groups because of the small sample size. After excluding those who died with head injuries, the mortality rate was lower in the conservative group than the open surgery group, but this difference was statistically non-significant (5.7% vs. 15.8%; *p* = 0.06).

A male preponderance (82%) was observed in our cohort population and road traffic accidents (64%) were the most frequent cause of BTAI. These findings are in-line with the earlier studies from other centers [16, 17]. The mean age of patients was 37.3 ± 14.5 indicating a young afflicted population as compared to an earlier study from the United States which reported higher mean age of 46 ± 20 years [18].

Historically, open surgical repair of BTAI carries a higher rate of mortality (28%) and paraplegia (16%) [19]. With increased CT scan imaging, the diagnosis of BTAI at our institute increased and also the paradigm shift in the management from open surgery to a minimally invasive modality (TEVAR) over the years.

In our study, the TEVAR group was relatively younger with a male predominance and no stent-related complications were observed. Notably, young patients tend to have a more acute curvature of the aortic arch hence an increased risk of subsequent endoleak and stent-graft collapse [20]. Also, the mortality in the TEVAR group (6%) was significantly lower than the open surgery group (31.6%). A prospective, multicenter study that compared operative repair to Stent repair showed that the TEVAR group had a significantly lower mortality (aOR: 8.42) and fewer blood transfusions (adjusted mean difference: 4.98) [21]. Two meta-analyses on BTAI found that endovascular repair outperformed surgical repair in terms of mortality [22, 23]. However, a recent meta-analysis found that postoperative mortality was not significantly different between the two groups but TEVAR was associated with a reduced paraplegia rate compared to open surgery [24]. An earlier study reported that postoperatively the rate of 30-days mortality and paraplegia was 10% each in the operative repair group. In contrast, lower rates (4.5%) were observed in the endovascular stent grafting group, which was not statistically significant [25]. In our cohort, postoperative complications were fewer as only one patient developed paraplegia due to ischemia and one developed deep vein thrombosis in the open repair group. Contrarily, these complications were not seen in the other groups. Paraplegia in the open repair group could be minimized by the maintenance of the distal perfusion during aortic cross clamping by active or passive shunting in most of the patients. A recent meta-analysis reported that the pooled prevalence of 30-day all-cause and aortic-related mortality was 2.2% and 2.1%, respectively [26]. In addition, the pooled prevalence rates of 30-day complications, such as type 1 endoleak, endograft complications, vascular access injury, strokes, and aortic re-rupture were 1.2%, 0.34%, 0.14%, 0.02%, and 0.01%, respectively.

Steuer et al. [20] studied long-term outcome in patients treated with TEVAR and reported 5-year survival rate of 81%. In contrast, survival was 94% in our cohort, with continuous clinical follow-up in some patients. The authors reported that the incidence of complications seems to be highest during the first year. The need for endovascular re-intervention or the occurrence of aortic-related death is much lower after the first year, suggesting an infrequent follow-up regimen over time.

Kokotsakis et al. [25] reported that the main Endovascular prerequisites are proximal and distal landing zone of at least 1–2 cm length for the left subclavian artery was deliberately covered in 2 patients to increase the length of the proximal landing zone. In our study, intentional partial left subclavian artery coverage was done for a short proximal landing zone. These patients remained asymptomatic without the need for revascularization, possibly due to good collateral blood supply. Scalloped endografts are also available to address the issue of a short proximal landing zone [27].

Usually BTAI with Grade II injuries and above have an indication for surgical intervention but interestingly all our patients with Grade I-II injuries were managed conservatively. Moreover, a higher proportion of grade III patients survived while mortality in our study was significantly higher in grade IV patients. None of the stented patients underwent a secondary surgical or endovascular procedure, which indicates appropriate selection and management of BTAI.

Although the decision on the type of intervention becomes clearer now, the timing of interventions is still not well defined due to the lack of clinical guidelines. The Society for Vascular Surgery recommends urgent repair (within 24 h) or after other injuries have been stabilized, observation of minimal aortic defects, selective versus routine revascularization in cases of left subclavian artery coverage, and that spinal drainage is not routinely required in these cases [13]. A prospective study compared early repair (≤ 24 h) versus delayed repair groups (> 24 h) reported that the delayed repair of stable BTAI patients was associated with improved survival, irrespective of the presence or absence of major associated injuries [28]. Estrera et al. reported that the delayed repair was done in 41% of cases and was associated with only 1 death (2%), significantly lower than immediate repair with 28% mortality [29]. Another study by Spiliotopoulos et al. showed that the midterm outcomes of TEVAR for patients with stable repair after BTAI were excellent, in terms of short (1.0–1.5 years) and long-term (> 1.5 years) follow-up after a median surveillance period of 3 years [30]. All the 76 implantations in that study were done by the interventional radiologists as opposed to the current study that mainly involved trauma and vascular surgeons for all the procedures.

In our study, the overall mortality in patients with BTAI was related to associated head injuries, high ISS, and high aortic injury grade. These findings are consistent with a previous observational study that found a substantial connection between high ISS, hemodynamic instability, and the requirement for vasopressors and death [18].

### Limitations

The retrospective design and single-center data are the inherent limitations of the present study. Moreover, the sample size may limit the generalizability of the findings. However, this incidence of BTAI could be underestimated as the study did not consider those who died on arrival and postmortem examination was lacking for the prehospital deaths. Moreover, this sample could be representative of the country as our center is the only tertiary facility that deals with traumatic aortic injuries in Qatar. In addition, the dataset did not provide time intervals from injury to interventions. Despite these limitations, the current study provided a well-described overview of the management options and consequences of BTAI at a trauma center in a rapidly developing Middle Eastern country.

In **conclusion**, BTAI is not common in our trauma patients, however, one-quarter of cases died in a level 1 trauma center, prehospital deaths were not analyzed, and no postmortem examination was performed. The associated TBI and aortic injury grade have an impact on the management option and hospital outcome. The conservative and TEVAR options were performed almost equally in 78% of cases. TEVAR and open surgery were performed only for aortic injury grade III or IV whereas the conservative treatment was offered in selected cases among the 4 injury grades. However, the mortality was higher in the conservative followed by the open surgery group and mostly due to the associated severe head injury. TEVAR should be offered to patients of BTAI deemed for intervention unless contraindicated due to technical difficulties. TEVAR does not need an intrusive thoracotomy/sternotomy, aortic clamping, or cardio-pulmonary bypass, as these procedures can be hazardous in a critically ill patient. However, it offers a quick reinforcement of the injured aortic wall. The blood loss and the possibility of spinal cord injury are minimized. Moreover, the in-hospital mortality is comparatively less with better short-term outcomes. Appropriately selected patients with low-grade injuries may be managed non-operatively. Finally, long-term follow-up is needed in young adults for concerns of aortic remodeling and complications.

## Declarations

### Availability of data and materials

All relevant data were given in the result, tables and figures in this manuscript.
